# Spatial structure of TLR4 transmembrane domain in bicelles provides the insight into the receptor activation mechanism

**DOI:** 10.1038/s41598-017-07250-4

**Published:** 2017-07-31

**Authors:** Konstantin S. Mineev, Sergey A. Goncharuk, Marina V. Goncharuk, Pavel E. Volynsky, Ekaterina V. Novikova, Alexander S. Aresinev

**Affiliations:** 1 Shemyakin-Ovchinnikov Institute of Bioorganic Chemistry, Mikluho-Maklaya 16/10, Moscow, 117997 Russian Federation; 2 Lomonosov Moscow State University, Moscow, 119991 Russian Federation; 3 Moscow Institute of Physics and Technology (State University), Institutskiy Pereulok 9, Dolgoprudny, Moscow Region, 141700 Russian Federation

## Abstract

Toll-like receptors (TLRs) play a key role in the innate and adaptive immune systems. While a lot of structural data is available for the extracellular and cytoplasmic domains of TLRs, and a model of the dimeric full-length TLR3 receptor in the active state was build, the conformation of the transmembrane (TM) domain and juxtamembrane regions in TLR dimers is still unclear. In the present work, we study the transmembrane and juxtamembrane parts of human TLR4 receptor using solution NMR spectroscopy in a variety of membrane mimetics, including phospholipid bicelles. We show that the juxtamembrane hydrophobic region of TLR4 includes a part of long TM α-helix. We report the dimerization interface of the TM domain and claim that long TM domains with transmembrane charged aminoacids is a common feature of human toll-like receptors. This fact is analyzed from the viewpoint of protein activation mechanism, and a model of full-length TLR4 receptor in the dimeric state has been proposed.

## Introduction

Toll-like receptors (TLRs) play a key role in the innate and adaptive immune systems^1^. TLRs share the common architecture of type I transmembrane proteins and comprise the extracellular ligand-binding domain (ECD), single transmembrane α-helix and intracellular Toll-interleukin I receptor domain (TIR), which is responsible for the downstream signaling^1, 2^. According to the X-ray crystallography and functional studies, TLRs form homo- or heterodimeric signaling complexes, interacting with pathogen-associated molecules^3–7^. The medical and biological significance of TLR signaling is obvious, since the dysregulation of TLR system may cause various autoimmune diseases and septic shock^8–11^, and some therapeutic strategies targeting TLRs have already emerged^8, 11, 12^.

Unlike many other type I membrane proteins, such as receptor tyrosine kinases (e.g. EGFR), which are dimers in both the active and inactive states, TLRs are thought to be activated via the “ligand-induced dimerization” mechanism; dimerization is usually accompanied by the migration to specific microdomains of cell membrane^13^. The only exception is the TLR9 receptor, which was shown to exist as preformed dimers in the cell membrane, and ligand binding induces only the rearrangement of its subunits^14^. While a lot of structural data is available for the ECD and TIR domains of TLRs, and a model of the dimeric full-length TLR3 receptor in the active state was build^6^, the conformation of transmembrane domain (TMD) and juxtamembrane regions in TLR dimers is still unclear. Only the structure of TLR3 isolated TMD dimer in detergent micelles was reported recently^15^. However, the importance of both receptor regions for the TLR activation is obvious, according to the recent studies^13, 16^. In particular, it was shown that decoupling of the ECD and TIR domains of TLR4 from the TMD can disrupt its signaling^17^ and that TLR4 constructs with deleted ECDs are constitutively active and dimeric^18^. Polymorphism (I602S) in the TMD of TLR1 was shown to correlate with the modified immune response to tri-acylated lipopeptides, suggesting that this region might be involved in the regulation or activation of the TLR1/2 complex. Besides, this polymorphism is also associated with the Crohn’s disease^13, 19, 20^. Isolated TMDs of all TLR receptors were shown to homodimerize in bacterial membranes, with TMDs of TLR2,3,8,9 demonstrating the highest propensity to take part in homotypic interactions^21^. Finally, the TMD of TLR2 and peptides, engineered based on the TMDs of TLR2 and TLR6, were shown to inhibit the full-length TLR2 receptor^13^, resulting in increased survival of mice with sepsis. On the other hand, it is noteworthy that TMD sequences of TLRs are rather peculiar for type I membrane proteins for two reasons: (1) they are characterized by almost zero homology for most TLRs and (2) they do not contain any wide-spread helix-helix interaction motifs, such as “glycine zipper” or “heptad repeat”^22^. With all aforesaid, it is obvious that structural investigations of TLR TMD dimers are necessary, because these domains can serve as targets for emerging therapies.

Apart from the TMD, deletion of the short hydrophobic intracellular linker region (ICL), connecting the TM and TIR domains of TLR4 can inactivate the receptor and alter its oligomerization propensity^23, 24^. The ICL region of TLR4 contains a patch of hydrophobic residues, which was referred to as the HR (hydrophobic region) domain. The recent work suggests that the HR domain contains a number of potential cholesterol-binding motifs and can be utilized to transfer the receptor between the liquid crystalline membrane and ordered microdomains upon the ligand binding^16^. Moreover, the specified transfer of protein was suggested to be accompanied by the elongation or shortening of the receptor TMD due to the interaction with cholesterol and thicker bilayer, which is observed usually in membrane rafts. In order to assess the possibility of such process and look at the structural organization of the juxtamembrane and TM portions of the TLR4 receptor, we investigate here the structure of TLR4 TMD in both the absence and presence of ICL in a variety of membrane mimetics, including the phospholipid bicelles.

## Results

### The hydrophobic part of TLR4 ICL is helical

**Figure 1:**
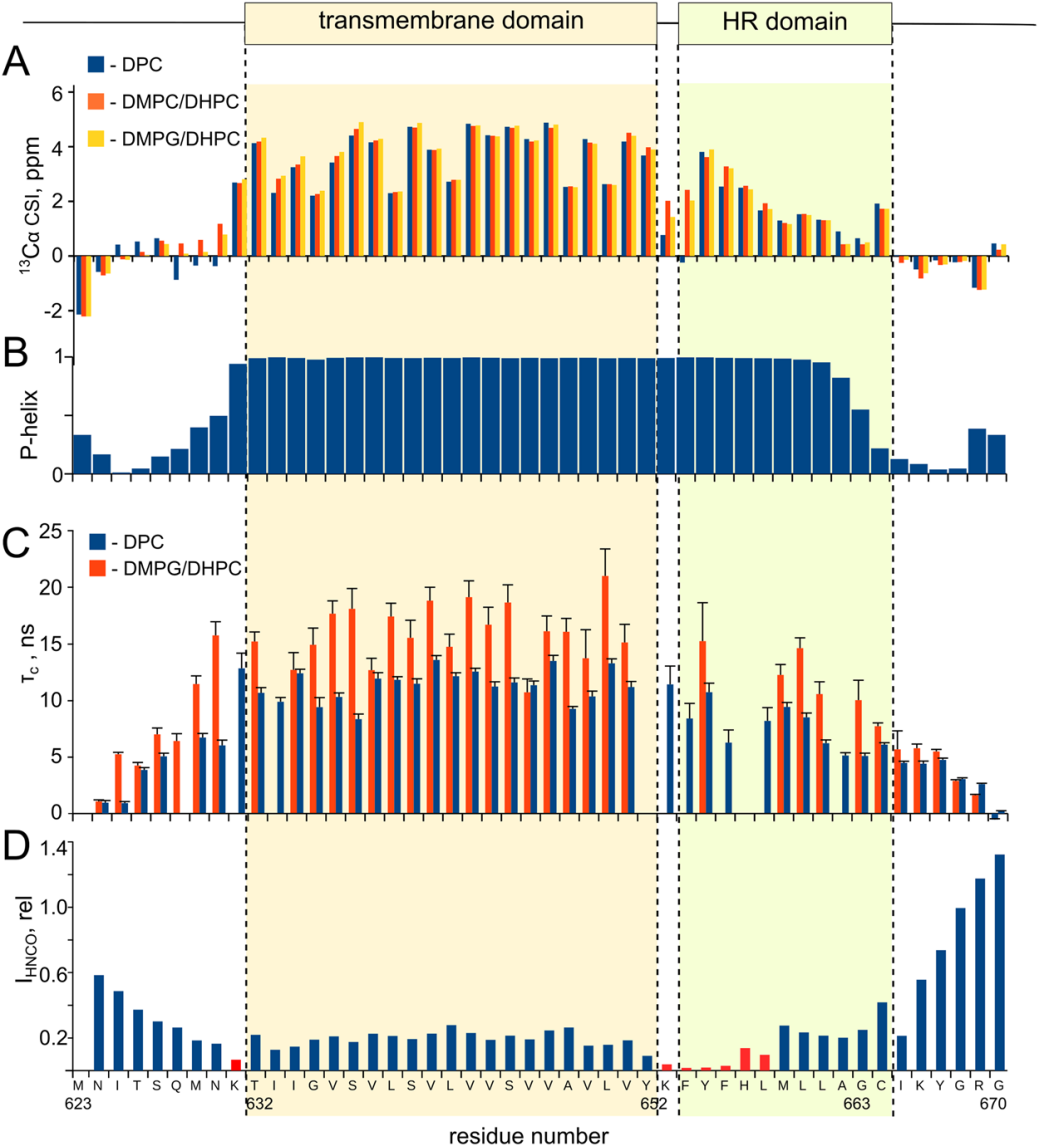
Secondary structure and dynamics of TLR4-TMICL. (**A**) Secondary chemical shifts of Cα nuclei of TLR4-TMICL in the environment of DPC micelles (blue bars), DMPC/DHPC q = 0.4 bicelles (red bars) and DMPG/DHPC q = 0.4 bicelles (yellow bars). Positive values indicate the helical conformation. (**B**) Propensity of helical secondary structure for the residues of TLR4-TMICL in DMPG/DHPC bicelles, according to the chemical shift based prediction by TALOS-N^52^. (**C**) Correlation time of rotational diffusion measured for the individual amide groups of TLR4-TMICL in DMPG/DHPC q = 0.4 bicelles (red bars) and DPC micelles (blue bars). Correlation times were measured from the rates of cross-correlated relaxation^55^. (**D**) Cross-peak intensities in the 3D HNCO spectrum of TLR4-TMICL in DMPG/DHPC q = 0.4 bicelles, expressed in relative units. Low intensity indicates the line broadening, enhanced transverse relaxation and motions in us-ms timescale. On top, the predicted TM domain (according to UniProt) and hydrophobic part of the ICL region (HR domain), homologous to the reported in ref. 23 are indicated.

To study the structural organization of TM and juxtamembrane parts of TLR4 we produced a receptor fragment (624–670), containing the TLR4 TMD, according to the Uniprot database (632–652), and whole intracellular linker region (653–670), named TLR4-TMICL. TLR4-TMICL was then incorporated into the detergent micelles (DPC) and DMPC/DHPC bicelles to assess its intramolecular mobility and spatial structure (Figure S1A–C). Both samples were investigated by solution NMR, which allowed the measurement of backbone and side chain chemical shifts that were afterwards utilized to describe the protein secondary structure. Surprisingly, chemical shift data reveal the helical structure of TLR4-TMICL on the region 631–663, which includes both the predicted TMD and an HR part of the ICL. (Fig. 1A,B). No sign of loss of α-helical conformation is observed on the border between the TM and ICL regions except for the chemical shifts of F^654^ which are close to the random coil conformation in micelles but not in bicelles. Since it is well known that the inner leaflet of cell membrane is negatively charged and TLR4-TMICL construct contains the positively charged K653 side chain immediately after the presumable TMD, we proposed that the anionic lipids could affect the behavior of the protein, and incorporated the TLR4-TMICL into the anionic DMPG/DHPC bicelles at neutral pH^25^. In the environment of anionic lipids the ICL region of TLR4 retained its helical structure, and chemical shifts of the whole region 631–663 corresponded to the straight α-helix conformation.

### ICL contains a part of the TLR4 transmembrane domain

**Figure 2:**
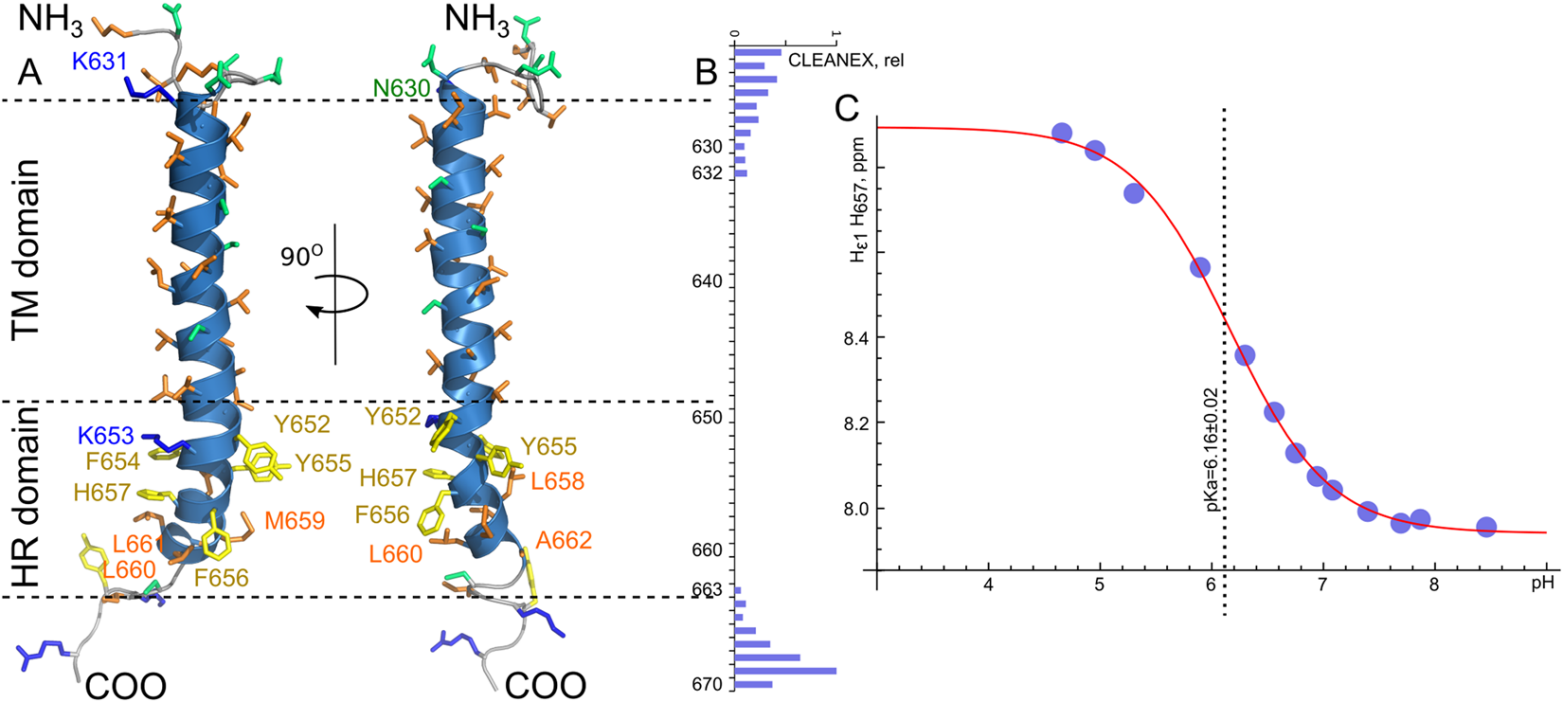
Spatial structure of TLR4-TMICL. (**A**) NMR-derived spatial structure of TLR4-TMICL in DMPG/DHPC q = 0.4 bicelles, pH 6.0, LPR 200. Residues in the hydrophobic part of the ICL region of the protein are indicated and painted according to their physical properties: Hydrophobic by orange, aromatic by yellow, polar by green and positively charged – by blue. (**B**) relative rates of the amide protons exchange with the solvent, as measured in the CLEANEX experiment^26^ for the TLR4-TMICL in DMPG/DHPC q = 0.4 bicelles at pH 7.2. (**C**) Dependence of the Hε1 chemical shift of H657 on the ambient pH which was used to determine the corresponding pKa. The fit of the obtained data to the equation (1) is shown by the red solid line.

Samples of TLR4-TMICL were unstable in zwitterionic lipids at neutral pH, therefore we used the TLR4-TMICL monomer sample in DMPG/DHPC bicelles to resolve the spatial structure of the protein. To do so, we utilized the chemical shift data and two 3D NOESY-HSQC spectra, which allowed obtaining 291 distance and angle restraints (Table S1). Finally, the spatial structure of TLR4-TMICL was determined in anionic lipids, which confirmed our preliminary data. The protein chain folded into the long 33-residue helix on the region 631–663, flanked by the short terminal disordered regions, without any sign of kink in the region of residues 653–657, which contains the polar K and H side chains (Fig. 2A). Structure of the protein is defined rather poorly on the specified region and the number of backbone-backbone NOE contacts is relatively low (Figure S2) due to the line broadening of signals, corresponding to the backbone nuclei of Y652,K653, F654, F656 and H657. However, several side chain-side chain contacts were observed, which are in agreement with the helical conformation within the region. In particular, methyl groups of L650 are in contact with the aromatic ring of F654; methyls of V651 – with the ring of Y655; methyls of L660 – with the ring of F656, and methyls of L658 – with the ring of F654 (Figure S3). Thus, there is a network of side chain-side chain contacts formed in the interfacial region between the TM and ICL parts of TLR4-TMICL with the step of 4 residues, characteristic for the helical structure.

To further investigate the TLR4-TMICL construct in the bilayer environment and assess its intramolecular dynamics, we measured the ^1^H,^15^N cross-correlated relaxation rate and calculated the correlation times of rotational diffusion for the protein N-H groups. Analysis of intramolecular dynamics as well reveal that no fast motion is observed within the long helix, and motions of the helical part of ICL region occur with the same characteristic time as the motions of TMD (Fig. 1C). In other words, TM and ICL segments are moving cooperatively and are within the same element of secondary structure – long α-helix. Thus, the HR segment of ICL is a part of the long TM domain of TLR4. This last conclusion is in agreement with the sequence of the protein, the ICL does not reveal any significant amphipathy, which is common for the juxtamembrane helices, associated with the membrane surface. Twelve ICL residues in a row (F654-I666) are highly hydrophobic, and definitely need to be membrane-embedded.

There are also other indirect data, confirming the transmembrane position of the HR part of ICL. First, all amide protons of the helical part of ICL are not accessible for the water, according to the CLEANEX experiment, which measures the rate of the amide proton exchange with the solvent^26^ (Fig. 2B). Second indicator is the side chain of H657. Random-coil pKa of His side chain is 6.8–6.9^27^. A similar value was reported for the H724 side chain of TLR3 in DPC micelles^15^, which is located in the last turn of the TM helix. If H657 side chain is close to the anionic lipid headgroups and is engaged in the ionic interactions, we expect its pKa to be elevated, because the ionized state is more favorable. In this manner, pKa of His50 of α-synuclein is increased by 1.2 pH units, when the protein is studied in the presence of anionic SDS micelles^28^. On the other hand, if the residue is inside the membrane, the neutral state is favorable and pKa has to be reduced. According to theoretical calculations, histidine pKa may reach 4.0 if the side chain is located close to the middle of the lipid bilayer^29^. In our case, the pKa of H657 is reduced to 6.16 ± 0.02 (Hill’s coefficient is equal to 0.98 ± 0.06), according to NMR measurements, which indicates that the residue is membrane-embedded and is not close to the lipid headgroups region (Fig. 2C). However, we have to point out that the presence of charged lysine side chain in the close proximity to the histidine residue is another factor that can shift the pKa and favor the uncharged state of imidazole ring.

**Figure 3:**
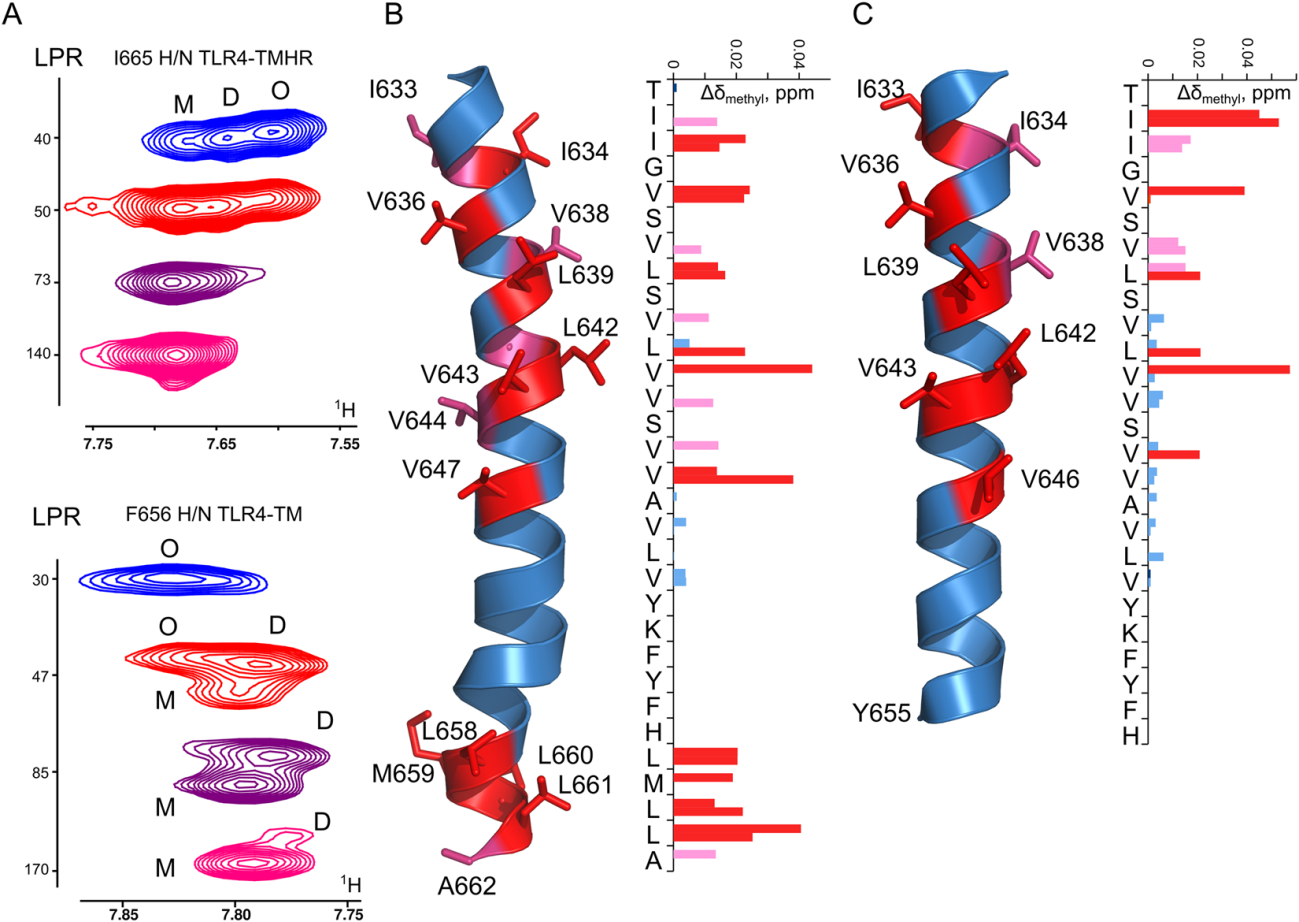
Dimerization of TLR4 TM domain in DPC micelles. (**A**) Fragments of the ^1^H,^15^N-TROSY spectra of TLR4-TMICL (I665, above) and TLR4-TM (F656, below) in DPC micelles, recorded at various LPR, as indicated. Peaks, corresponding to the monomeric (M), dimeric (D) and oligomeric (O) states are indicated. (**B**,**C**) Chemical shift variations of the methyl groups of TLR-TMICL (B) and TLR-TM (C), occurring upon the dimerization. Side-chains with generalized chemical shift changes exceeding 0.02 ppm are shown in red, and side chains with generalized chemical shift changes between 0.01 and 0.02 are shown in pink.

For comparison, we also produced a construct, containing the TM domain of TLR4 with the truncated ICL region (residues 624–657, TLR4-TM). This protein was studied in DPC micelles, and the helical conformation was shown to be adopted by residues 632–655, and is not distorted for the interfacial residues K653-Y655 (Fig. 3C).

With all aforesaid, we can state that the HR portion of ICL is a part of TLR4 TM domain in DMPG and DMPC bilayer. However, NMR data reveal that region 652–657 is mobile in the ms-us timescale – corresponding signals in ^15^N-HSQC and HNCO spectra are broad (Fig. 1D). The effect is pH-dependent, and is more pronounced at neutral pH, while narrower peaks are observed at pH below 6.0. Most likely, the helical conformation is more stable, when both the K653 and H657 side chains are charged – they are located on the same face of the helix, and electrostatic repulsion may favor the long helix structure. Thus, while on average a straight helix is formed, there is some flexibility between the predicted TM and ICL domains, which implies the possibility of other structures in thin bilayers or in the presence of specific lipids.

### Dimerization surfaces in TLR4-TM and TLR4-TMICL

To further investigate the structure of TM and ICL domains of TLR4, we studied the dimerization of two above mentioned TLR4 fragments. TLR4-TMICL was stable in DMPG/DHPC bicelles at high lipid-to-protein ratio, LPR, while when we try to saturate bicelles and reach the lower LPR values (ca. 100), the protein starts to precipitate and no dimeric state is observed. In turn, TLR4-TM is unstable in all types of bicelles and the sample lifetime does not exceed 3–4 days. Therefore, the dimerization of proteins was studied in DPC micelles, in this environment low LPR values can be obtained and the helical structure of TLR4-TMICL is retained, according to NMR chemical shifts. Both TLR4 fragments revealed the presence of three oligomeric states – monomer, dimer and trimer, depending on the LPR (Fig. 3A). Using our previously published approach^30^, we estimate the free energy of dimerization for TLR4-TM as −1.8 kcal/M (however, we need to point out that this number is a rough estimate, since we are not able to quantify properly the population of high-order oligomer). TLR4-TMICL behaved non-ideally in DPC and at LPR 100 almost no dimeric state was observed, which prevents the free energy calculation. Due to the presence of high-order oligomer, the population of dimeric state did not exceed 50%, and, since the major part of TMD is composed of valine residues, we registered highly overlapped ^13^C-HSQC spectra. These two obstacles hinder the direct solution of dimeric conformation, however, some conclusions can be drawn based on the chemical shift changes, occurring upon the dimerization of constructs. Amide groups are not suitable as the indicators of dimerization interfaces in membrane proteins, because they to a high extent depend on the hydrogen bond length; and their chemical shifts are perturbed mainly due to the slight changes in helical structure – bending, twisting or stretching of α-helices. In many documented cases, amide chemical shifts change throughout the whole TM helix upon the dimerization^15, 31^, which implies that methyl groups need to be considered as a source of data about the dimer structure. Using high-resolution 3D NMR experiments with constant time evolution of ^13^C magnetization^32^ and taking into account the dependence of monomer and dimer populations on LPR, we managed to assign the methyl signals of almost all residues in both monomeric and dimeric states of TLR4-TM and TLR4-TMICL in DPC micelles (Figure S4). Both TLR4 fragments reveal the chemical shift changes that are located mainly on the same face of the TM domains. The largest chemical shifts differences are observed for the methyl groups of I634, V636, L639, L642, V643, V646, V647, and residues L658-L661 in the HR part of TLR4-TMICL (Fig. 3B). This is a non-polar and very long interface containing the aromatic residues F654-Y655. Analysis of correlations between the chemical shift changes occurring upon the dimerization of TLR4-TM and TLR4-TMICL reveals that most changes are similar, while in case of TLR4-TM the methyl groups of I633, V636 and V646 are more sensitive to the dimerization, implying that the dimer contact area is shifted towards the N-terminus of the TM helix (Fig. 3C). Therefore we conclude that both the TLR4-TMICL and TLR4-TM dimerize weakly and via the long hydrophobic surface in the environment of DPC micelles, which is located on the same side of the TM helix, and hydrophobic region of intracellular linker, which is a part of the actual TLR4 TM domain, is involved in the dimerization. The indicated interface may correspond to the active state of the full-size TLR4.

### Model of the TLR4 TMD dimer

**Figure 4:**
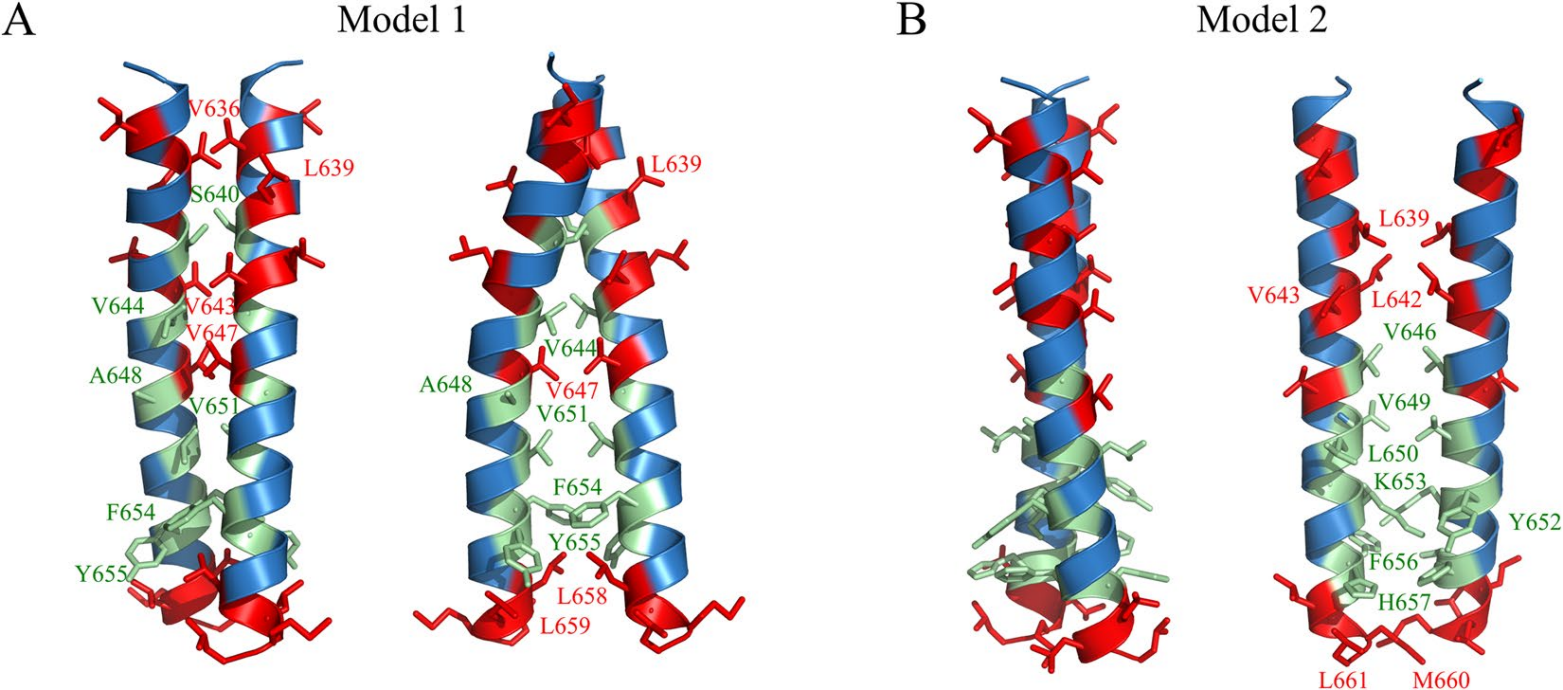
Two top-ranked dimeric conformations of TLR4-TMICL in DPC micelles out of 12 possible structures, generated by TMDOCK^33^. Residues, experiencing the maximal chemical shift perturbations upon the dimerization are shown in red. Other residues on the dimerization interface are shown in pale green and assigned. Model 1 (**A**) was selected as the most probable conformation of TLR4-TMICL dimer, while the Model 2 (**B**) was rejected due to the inconsistency with NMR data.

The reported chemical shift changes can be used to build a model of TLR4 TMD dimer, however, this is not a straightforward task. Not all residues in the TLR4-TMICL construct have methyl groups, therefore, the sampling of helical surface is not complete. Additionally, chemical shifts of some methyl groups can be perturbed upon the dimerization due to the changes in the structure of helix that take place, but not because these groups are located on the dimer interface. Therefore, we cannot directly transform our chemical shift data into the distance restraints and perform the structure calculation, this could result in the wrong conformation. To solve this problem, we used the docking approach. We utilized the TMDOCK software, which was published very recently^33^ and was shown to predict well the NMR-derived spatial structures of transmembrane dimers. The program generated 12 possible low-energy models of TLR4-TMICL dimers, which is in agreement with our observation that TLR4-TMICL has several surfaces for the helix-helix interactions, resulting in the presence of oligomers in solution. The obtained structures were ranked, based on the buried solvent accessible surface area of residues with perturbed chemical shifts of methyl groups (shown in red in Fig. 3). This procedure led us to two possible dimer conformations that at best fit to NMR data (Fig. 4).

The top-ranked model has four residues with large chemical shift deviations on the dimerization interface: V636, L639, V643 and V647, three of them were selected by TMDOCK as members of the main dimerization motif. Full motif has the following sequence: ^636^ **V**xx**LS**xx**V**VxxVVxxVxxFYxxLL^659^ (Fig. 4A). The dimer is right-handed, the axes of TM helices cross at 28° at the closest distance of 7.9 Å. The interface is extended and takes almost the whole length of TMD helix. Helix-helix interactions are supported by side chain hydrogen bonds formed by S640 and π-stacking of F654 and Y655. The second model places only V639, L642 and V643 on the dimerization interface (Fig. 4B). This dimer is left-handed with almost parallel arrangement of TM helices (12°), and large distance between the helical axes, 10.1 Å. The dimerization interface includes the following residues: ^639^VxxLVxx**V**xxVLxY**K**xxFHxx**M**L^661^, it is shifted towards the C-terminus of TMD and key residues, according to TMDOCK are V646, K653 and M660. The conformation is stabilized by the π-stacking of aromatic rings and π-cation interactions formed by the side-chain of K653. However, such dimerization interface implies the tight contact between the K653 side-chain and aromatic residues, which should result in the chemical shift perturbations of K653 CεH_2_ group, which is not observed in NMR spectra upon the dimerization of TLR4-TMICL in DPC micelles (Figure S5). Thus, we conclude that the second model is not supported by NMR data, and the right-handed model shown in Fig. 4A corresponds to the conformation of TLR4-TMICL dimer in DPC micelles.

## Discussion

### All TLR members have hydrophobic ICLs which can contain the parts of their TM domains

The original article^23^ reports the hydrophobic ICL as a specific feature of murine TLR4. It also reports several major effects of the ICL on the behavior of full-size receptor: deletion of the hydrophobic part of ICL impairs the oligomerization of the receptor and ability of TLR4 to induce the inflammatory response. The work was performed using the murine TLR4 which does not contain the charged residue, homologous to the K653 of human receptor, and the whole TMD-ICL part of murine protein is highly hidrophobic. In other words, there is no reason, why the ICL and TM domains were divided when annotating the murine TLR4, looking at the sequence of the protein, it is obvious that the ICL contains a part of protein TM domain. The later work by Daringer *et al*.^24^ studied the human variant of TLR4 and also found the adverse effects of the ICL. Deletion of the linker or, on the contrary, its elongation or substitution with various sequences was found to cause the unpredictable effects on the LPS-mediated signaling by TLR4. Reversing or scrambling of the ICL hydrophobic sequence did not abolish the LPS-mediated TLR4 signaling. Moreover, ICL can be partially substituted on polyglycine or some arbitrary positively charged sequence without the complete loss of activity, while slightly polar and negatively charged sequences inactivated the receptor. However, in all artificial constructs the activity of TLR4 was reduced. These facts indicate that almost no specificity is encoded in the ICL, and its hydrophobicity is not a prerequisite for the signaling to take place.

**Figure 5:**
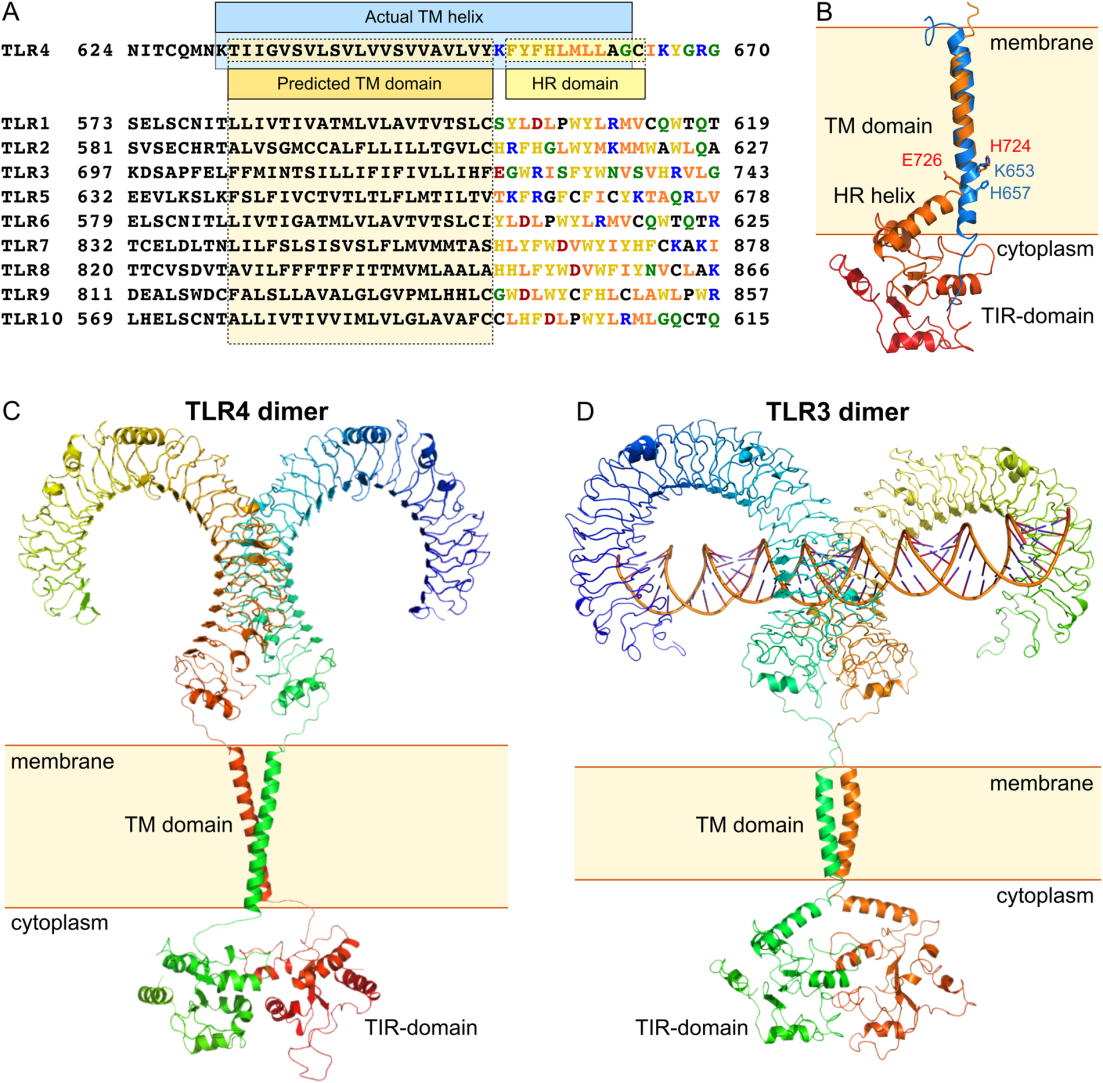
TM domains of human toll-like receptors. (**A**) Sequence alignment of human TLR members. Residues in the predicted cytoplasmic juxtamembrane regions are colored according to their physical properties. Hydrophobic residues are shown by orange (ILVM), aromatic – by yellow (HWFY), positively charged – by blue, negatively charged – by red, polar – by green (GSQN), neutral – by black (APTC). The predicted (according to UniProt) TM domain and HR region, homologous to the reported by Nishiya *et al*.^23^ are indicated. (**B**) The reported structure of TLR4-TMICL (blue) is superimposed with the TM domain of TLR3 in the model of the full-size receptor (brown-red)^6^. Key H and charged residues on the interface between the predicted TM and ICL domains are indicated and signed. (**C**) Model of full-length TLR4 receptor, constructed based on the X-ray structures of ECDs and TIR domains and reported NMR data. (**D**) model of the full-length TLR3 receptor, proposed by Liu *et al*.^6^. Coordinates were provided by David Davies, NIH.

Daringer *et al*. had also argued that the hydrophobicity of the intracellular linker region is a unique feature of TLR4 and other TLRs are characterized by polar juxtamembrane regions. We claim that it is not correct. The analysis of the TM and juxtamembrane parts of human TLRs reveals that ICL of TLR4 has nothing peculiar in its aminoacid sequence (Fig. 5A). All TLRs have 10–15 residues after the predicted TM domain, which are highly hydrophobic or aromatic and include 1–2 charged aminoacids. The hydrophobicity of ICL residues can be quantified using the whole residue scale, suggested by White and Wimley^34^, which describes the free energy of transfer of aminoacids between the polar (water) and non-polar (octanol) phases, results of the analysis are summarized in Table 1. As a measure of hydrophobicity, we used the average free energy of transition to the non-polar phase of first 11–16 residues after the predicted TM region, according to Uniprot. As a control, we calculated the average hydrophobicity of TLR4 TM domains (632–652), which was equal to −0.36 kcal/mol, and of the TIR domain (672–818), which was +0,38 kcal/mol. The hydrophobic part of TLR4 ICL, including the K653, was on average even more hydrophobic than its TM domain (−0.39 kcal/mol), and among other TLRs, TLR2 and TLR10 had the ICLs of similar hydrophobicity (−0.35–0.42 kcal/mol), while ICLs of TLRs 7,8 and 9 were much more hydrophobic (~−0.65 kcal/mol) and ICLs of TLR1,3,5 and 10 were slightly hydrophobic. Still, none of the studied ICLs is polar. For comparison, we estimated the hydrophobicity of amphipathic juxtamembrane helices of EGFR and HER2 receptors^35, 36^, and found out that they are highly hydrophilic despite their proved membrane activity (+0.83–0.89 kcal/mol).

**Table 1:** Hydrophobic properties of TLR juxtamembrane regions.

Protein	Resiue numbers^a^	Sequence	Hydrophobicity, kcal/mol^b^	Per residue
TLR4 TM	632–652		−7.56	−0.36
TLR4 TIR	672–818		55.9	0.38
TLR4 HR	653–665	KFYFHLMLLAGCI	−5.13	−0.39
TLR1^c^	602–614	SYLDLPWYLRMVC	−2.34	−0.18
TLR2	610–625	HRFHGLWYMKMMWAWL	−6.72	−0.42
TLR3	726–738	EGWRISFYWNVSV	−0.28	−0.02
TLR5	661–671	TKFRGFCFICY	−0.99	−0.09
TLR6	608–619	YLDLPWYLRMVC	−2.82	−0.24
TLR7	861–873	HLYFWDVWYIYHF	−8.7	−0.67
TLR8	849–860	HHLFYWDVWFIY	−7.99	−0.66
TLR9	840–856	GWDLWYCFHLCLAWLPW	−10.58	−0.6
TLR10	598–610	CLHFDLPWYLRML	−4.5	−0.35
EGFR JMA^d^	676–686	KRTLRRLLQER	10.94	0.99
HER2 JMA	683–696	RKYTMRRLLQETEL	11.63	0.83

^a^Residue numbering is given according to Uniprot.

^b^Hydrophobicity is a sum of contributions of distinct residues, according to ref. 34.

^c^Here and below shown are the first 11–16 residues immediately after the TM domain of corresponding TLR, until the encounter of first polar aminoacid.

^d^Cytoplasmic juxtamembrane helix of the EGFR and HER2 proteins.

Thus, all human toll-like receptors have similar ICL regions, which, as we report here on the example of TLR4, are likely to contain the part of their TM domains. All ICLs reveal no sign of amphipathy, which is a feature of short juxtamembrane helices that are present in some types of cell receptors. In other words, TLRs are characterized by relatively long (32–35 residues) TM helices with charged aminoacids quite deep inside the membrane. Both may be relevant for the functioning of receptors. The fact that ICL region contains a part of the TM domain explains all the findings reported by Nishiya *et al*.: deleting the part of the TM domain should result in the altered dimerization propensity of the protein, subcellular localization and certainly can impair the activation mechanics. It is also now obvious, that the length of the TM domain is not a necessary prerequisite of TLR signaling, since the TLR4 constructs with ICLs substituted with flexible linkers are still active^24^. However, the constructs with native ICLs provide the most intensive response to LPS among all tested variants, therefore, the native sequence is optimal for the signaling and ensures the correct compartmentalization of the receptor – TLR4 variants with scrambled or inversed ICLs are less likely to be present at the cell surface.

### ICL of TLR4 and cholesterol binding/raft localization

We would like to point out that we are not the first who noticed the homology and hydrophobicity in juxtamembrane regions of human TLRs. The 2015 paper by Ruysschaert and Lonez^16^ analyzed the known data about the TLR membrane localization and their aminoacid sequences and suggested the activation mechanism, involving the migration of TLRs to membrane microdomains and clusterization, triggered by the ligand binding and supported by the cholesterol binding propensity of ICL regions. According to the authors, almost all juxtamembrane regions of human TLRs contain one or two CRAC/CARC cholesterol-binding consensus motifs. The proposed model also implies that inactive TLRs are in the liquid membrane and have short TM domains, corresponding to the predicted in UniProt annotation. Activated protein are supposed to have elongated TM domains, which now include the cholesterol-binding site, encoding their preference for the thicker bilayer of liquid-ordered membrane microdomains. The mechanics and driving forces of this transition were, however, not put forward. We suppose that our data can clarify this process. We report here that the TM helix of TLR4 is substantially longer than is expected for the single-span TM protein even in the environment of short-chain phospholipid and in the absence of cholesterol. Average length of TM domains of receptor tyrosine kinases is 27–29 residues (36–40 A, which corresponds to the headgroup-to-headgroup thickness of liquid bilayers of several kinds)^37^, while we observe a 33-residue helix (47 A). This difference between the length of average TM domain and the observed helix of TLR4 is in a good agreement with the difference observed in the thickness of lipid bilayer in the liquid and liquid-ordered phases, which equals 6 Å^38^. Thus, the length of the TM helix is an intrinsic property of the receptor and is not controlled by the membrane composition, which, in some aspect, contradicts the previously mentioned activation mechanism. Since we claim that the receptor TMD is long in both liquid membrane and microdomains, and has a clear preference for thick bilayers that are rich in cholesterol, we need to explain why TLRs are not found inside the raft fractions of cell membranes before the ligand stimulation and are found in rafts after the ligand binding^39^. Two reasons could be proposed for this phenomenon: first, in monomeric TLRs the extracellular and TIR domains may interact with the membrane, disturbing the liquid-ordered phase and preventing the raft formation around the TM helix. In this case, the ligand-induced dimerization of TLRs is supported by the tight interaction of both ligand-binding and TIR domains^40^, these domains do not contact any more with the membrane in dimers, and proteins migrate to the lipid rafts with thick bilayer and large amounts of cholesterol. Second, the tilt of the TM helix may somehow control the membrane localization of monomeric and dimeric TLRs. The average tilt angles of the TM helix with regard to the bilayer normal should be different for monomers and dimers, and it is known that the protein-cholesterol interaction for tilted and straight helices follows the different laws and requires different sequence context^41^. Thus, change of the TMD tilt may result in the altered mode of cholesterol binding by the ICL regions of TLRs and either migration of proteins to the rafts or protein clusterization and formation of rafts around them. However, we need to adopt that the last hypothesis is less realistic, because the tilt is the result of the hydrophobic mismatch effect, and the same effect should rather result in the protein migration to thick bilayer areas than in the tilted state of a TM helix. On the other hand, the monomeric TM helix can easily tilt in the bilayer, while it is difficult to imagine the tilted TM domain within the dimeric TLR protein – the tilt may cause the dimer asymmetry and interaction of ECD and TIR domains with the membrane. Thus, while TLR monomer can compensate for the hydrophobic mismatch by tilting, dimer would migrate to thicker bilayer or cause the immediate formation of surrounding microdomain in the cell membrane.

### The architecture of the TM domain within the full-size receptor

Taking into account all aforesaid, it is possible to speculate about the structural organization of the full-length TLR4. There exists only a single full-size model of TLR member – the one of TLR3 (Fig. 5D)^6^. It was build based on the crystal structure of the receptor extracellular domain in complex with double-stranded RNA fragment, the homology model of the TIR domain and a common sense view on the TM and juxtamembrane domains. The model suggests that the TM domain is extremely short (21 residues) and is followed by a short loop and a long juxtamembrane helix which is almost as long as the TM domain (19 residues). The interhelical loop takes place in the region which is homologous to residues Y652-K653 of TLR4 and the whole part of TLR4, including the regions, homologous to the TM and juxtamembrane helices in the full-length TLR3 model, are present in our TLR4-TMICL construct. According to the reported data, the specified model is incorrect, at least regarding TLR4, and TM and juxtamembrane parts of the receptor are arranged differently than was suggested (Fig. 5B). To illustrate the difference, we have built the model of full-size TLR4 receptor in the dimeric state (Fig. 5C). The model is based on the X-ray structure of TLR4 ECDs^7^ (PDB ID 3FXI), homology model of TIR domains dimer, based on the structure of TLR10 TIR domains^42^ (PDB ID 2J67) and our NMR data concerning the TMD and ICL region of TLR4 (Fig. 4A). In fact, it is the first model of full-length Toll-like receptor, which is based on the experimental data in all three domains of the protein.

According to our model, instead of two helices proposed by Liu *et al*.^6^, there is a single relatively long helix, finishing with G663, and a disordered region, connecting the TM helix with the TIR domain. This region is approximately 8 residues long, because the TIR domain starts with the residue 672, according to the homology model. Thus, instead of the rigid connection between the TM and TIR domains, rather flexible linkage is expected. Unfortunately, we also have to state that the present data means that our recently published spatial structures of TLR3 TMD dimer and trimer^15^ may be also irrelevant, since the work was done on TMD constructs, where the C-terminal residue was in the middle of the ICL region of receptor, therefore the TM domain is likely to be truncated.

## Conclusion

In the present work, we studied the transmembrane and juxtamembrane parts of human TLR4 receptor using solution NMR spectroscopy in a variety of membrane mimetics, including the phospholipid bicelles. We show that the juxtamembrane ICL region of TLR4 is helical and contains a part of long transmembrane α-helix. We report the dimerization interface of the TM domain and claim that long TM domains with transmembrane charged aminoacids are a common feature of human toll-like receptors. This fact was considered from the viewpoint of protein activation mechanism, and a model of the full-length TLR4 receptor in the dimeric state is proposed.

## Methods

### Construction of expression plasmids

The gene, encoding the predicted (according to UniProt) transmembrane and several juxtamembrane residues 624–657 of human TLR4 (MN^624^ITSQMNKTIIGVSVLSVLVVSVVAVLVYKFYFH, TLR4-TM) was amplified by PCR from four chemically synthesized oligonucleotides (Evrogen, Russia) partially overlapped along their sequences. The PCR products were cloned into pET22b vector using ligation by NdeI and HindIII restriction sites. The final construct was confirmed by DNA sequencing. The gene, encoding the TMD and ICL residues 624–670 of human TLR4 (MN^624^ITSQMNKTIIGVSVLSVLVVSVVAVLVYKFYFHLLMLAGCIKYGRG, TLR4-TMICL) was constructed in a similar manner.

### Gene expression

The 35-residue TLR4-TM and 48-residue TLR4-TMICL were expressed as a precipitate of the reaction mixture (RM) in continuous exchange cell free (CF) expression system^43, 44^. S30 CF extract from Rosetta(DE3)pLysS E. coli strain and T7 RNA polymerase were prepared by previously described protocol^45^. Optimal reaction conditions were provided using the homemade reactors based on the Mini-CECF-Reactor^45^ and membranes with molecular weight cut-off of 12.5 kDa. Preparative scale reactions (1–2 ml of RM) were carried out in 50 ml tubes. The optimal FM:RM ratio was 12:1 and RM contained 100 mM HEPES/0.83 mM EDTA/KOH at pH 8.0, 0.1 mg/mL folinic acid, 20 mM acetyl phosphate, 1.2 mM ATP and 0.8 mM each of G/C/UTP, 2 mM 1,4-dithiothreitol, 0.05% sodium azide, 2% PEG-8000, 20 mM magnesium acetate, 270 mM potassium acetate, 60 mM creatine phosphate, 1 mM each of 20 amino acid or 0.25% of 20 amino-acid mix (CIL), 1 tablet/50 mL complete protease inhibitor (Roche), 0.5 mg/mL E.coli tRNA (Roche), 0.25 mg/mL creatine kinase from rabbit muscle (Roche), 0.05 mg/mL T7 RNA polymerase, 0.1 U/mkL Ribolock (Fermentas), 0.02 mkg/mkL plasmid DNA, and 30% S30 CF extract. All reagents were provided by Sigma Aldrich, Germany, unless otherwise specified. Reactions were conducted overnight at 30 °C and 150 rpm in an Innova 44 R shaker (New Brunswick). 20-aminoacid mixture of ^13^C/^15^N-labeled aminoacids (CIL) was used to obtain uniformly ^13^C/^15^N-labled protein sample.

### Protein purification and solubilization

The protein precipitate was washed three times by buffer containing 50 mM Tris pH 8.0 and 100 mM NaCl. RNAse A (Fermentas) with final concentration of 20 mkg/mkl was added into the buffer at first washing step. After each step protein was centrifuged for 10 min at 18000 g at room temperature and supernatant aliquots were analyzed by 12,5% Tricine SDS-PAGE. Both proteins were purified by the size-exclusion chromatography in lauryl sarcosinate micelles. Detergent was removed either by the dialysis (in case of TLR4-TM) or by the TCA/Acetone extraction procedure. The obtained precipitate was dissolved directly by the solution of DPC-d38 (CIL) in 20 mM pH 6.0 phosphate buffer in case of TLR4-TM. TLR4-TMICL was first dissolved in hexafluoroisopropanol (HFIP) solution which was later supplied by the necessary amount of lipid/detergent in dry powder. The obtained mixture was diluted gradually by pure water until HFIP/water ratio reached 1:1. The solution was lyophilized and the powder was resuspended in the aqueous buffer (20 mM phosphate, pH 6.0–6.5). Several samples were prepared in various membrane mimetics, including DMPC/DHPC and DMPG/DHPC bicelles and DPC micelles. Lipid/protein ratio was varied in the range 30–200, q of bicelles was varied in the range 0.3–0.5. Typical concentration of protein was 0.5 mM.

### NMR Spectroscopy

All NMR experiments were run on 600 and 800 MHz Avance III spectrometers equipped with cryogenic triple resonance probes (Bruker Biospin, Germany) at 45 °C. The dimerization of TLR4-TM was analyzed through the procedure described in^30^. Populations of the states were determined from the intensities of 2D cross-peaks in BEST-TROSY spectra^46^ recorded with the relaxation delay of 0.8 s (selective excitation T1 for amide protons was estimated as 0.15 s). Assignment of chemical shifts was performed via the standard approach^47^ using the triple resonance and NOESY 3D NMR spectra. Non-uniform sampling in indirect dimensions^48^ and BEST^49^ pulse sequences were used to record the triple resonance spectra. Constant-time evolution versions of NOESY-^13^C-HSQC and HCCH-TOCSY experiments^32^ were used to assign the signals from the methyl groups of proteins under investigation. Spin-echo difference spectra were used to calculate vicinal J-couplings, depending on χ_1_ dihedral angle^50, 51^. Backbone torsion angles restraints were obtained from the NMR chemical shifts in TALOS-N software^52^. Spatial structures were calculated in CYANA 3.0 software^53^, the obtained data were visualized in the PyMOL Molecular Graphics System (Version 1.8 Schrödinger, LLC). To determine the pKa of His side chain, the dependence of Hε_1_ chemical shift on the pH was fitted to the equation:

1 $$\delta (CS)=({\delta }_{a}+{\delta }_{b}{10}^{n(pKa-pH)})/(1+{10}^{n(pKa-pH)}),$$

where δ(CS) is the current chemical shift of the nucleus, and δ_a_ and δ_b_ are chemical shifts of the nucleus in the protonated and the deprotonated states, respectively, and n is the Hill’s coefficient^54^. To study the water accessibility of amide protons, we used the CLEANEX experiment with 20 ms mixing^26^. Ratio of peak intensities in the CLEANEX spectrum and reference HSQC spectrum was considered as a measure of the exchange rate. To quantify the chemical shifts changes of methyl groups we calculated the generalized chemical shift change:

2 $$\delta =\sqrt{{\rm{\Delta }}\delta {H}^{2}+{\rm{\Delta }}\delta {C}^{2}/16}$$

where ΔδH and Δ*δC* are the proton and carbon chemical shift changes, expressed in ppm units.

TROSY-based experiment for the measurement of cross-correlated relaxation rate^55^ was recorded in all membrane mimetics to estimate the correlation time of rotational diffusion, τ_c_. Relative intensity of cross-peaks in HNCO spectrum was considered as a measure of cumulative transverse relaxation of all nuclei in the peptide bond to identify the conformational exchange processes occurring in the µs-ms timescale.

### Constructing the model of full-length TLR4

The model of TLR4 dimeric structure in the active state was generated based on three experimental structures. Dimeric ECDs (residues 27–627) are available in PDB (3fxi)^7^. Dimeric form of TM helixes (residues 623–670) was obtained using the reported NMR structure and TMDOCK server^33^. From the resulting set of TMDOCK-predicted dimer conformations, we chose the structure with the helix-helix interface, containing the maximal number of methyl groups that reveal the chemical shift changes due the dimerization of TLR4-TMICL in DPC micelles. Dimeric form of TIR domain of the receptor (residues 653–839) was generated by the homology using Modeller 8.2^56^ based on the structure of TIR domain of TLR10 receptor (PDB ID: 2j67)^42^ as a template. Alinement was done using the Clustal omega server^57^. All these protein fragments were overlapped by amino acid sequences. To obtain the receptor model we used the conformation for 623–627 segment from the 3fxi structure, as this fragment is disordered in the context of TLR4-TMICL construct. Then we aligned these reduced protein fragments along one axis, and rotated and moved them along it in a way that the distance between the neighboring residues became minimal. Then we joined the protein parts into the dimer and relaxed this structure using 5000 steps of steepest descent energy minimization. Calculations were performed in gromacs 4.6 software^58^ in Amber ff99SB-ILDN force field^59^.

### Accession numbers

Spatial structure and chemical shifts (in all three mimetics) of TLR4-TMICL were deposited to PDB (accession code 5NAM) and BMRB (accession code 34108). Spatial structure and chemical shifts of TLR4-TM in DPC micelles were deposited to PDB (accession code 5NAO) and BMRB (accession code 34109).

## Electronic supplementary material

Supplementary materials
