# Supplementary material for: Spatial structure of TLR4 transmembrane domain in bicelles provides the insight into the receptor activation mechanism
Source: Sci Rep. 2017 Jul 31;7:6864. doi: 10.1038/s41598-017-07250-4 (PMC5537299; doi:10.1038/s41598-017-07250-4)
Supplement: Supplementary file 1 — Supplementary materials [file 41598_2017_7250_MOESM1_ESM.pdf]

Spatial structure of TLR4 transmembrane domain in bicelles provides the insight into the receptor activation mechanism.

Konstantin S. Mineev, Sergey A. Goncharuk, Marina V. Goncharuk, Pavel E. Volynsky, Ekaterina V. Novikova and Alexander S. Arseniev

Supporting data.

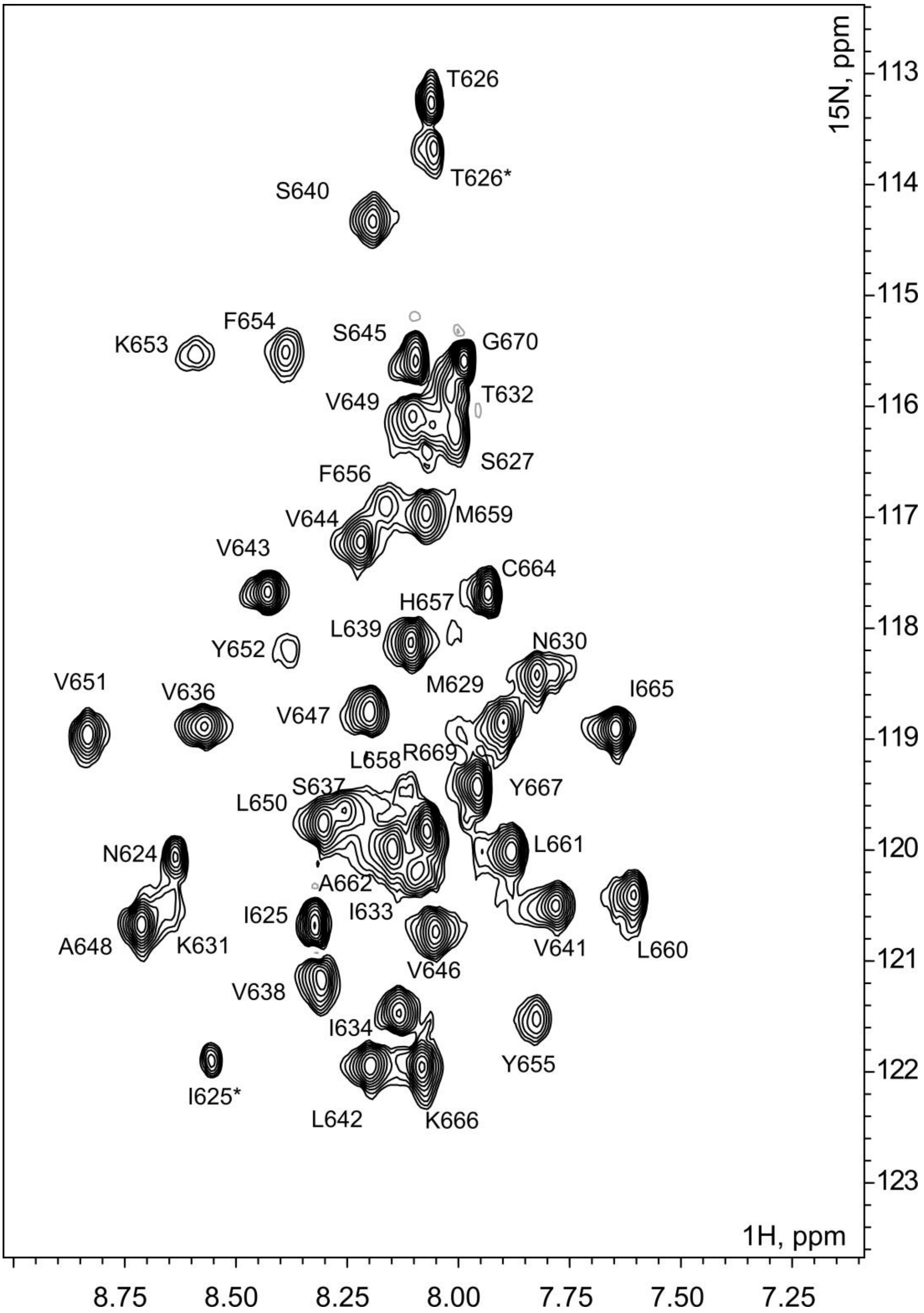

Figure S1A. Refers to Figure 1. Fragment of 1H,15N-BEST-TROSY spectrum of 0.5 mM TLR4-TMICL in DPC micelles, recorded at pH 6.0, 40 ºC, LPR 140. * denotes the minor state which is present at the N-terminus of the protein due to the partial formylation of the N-terminal residue.

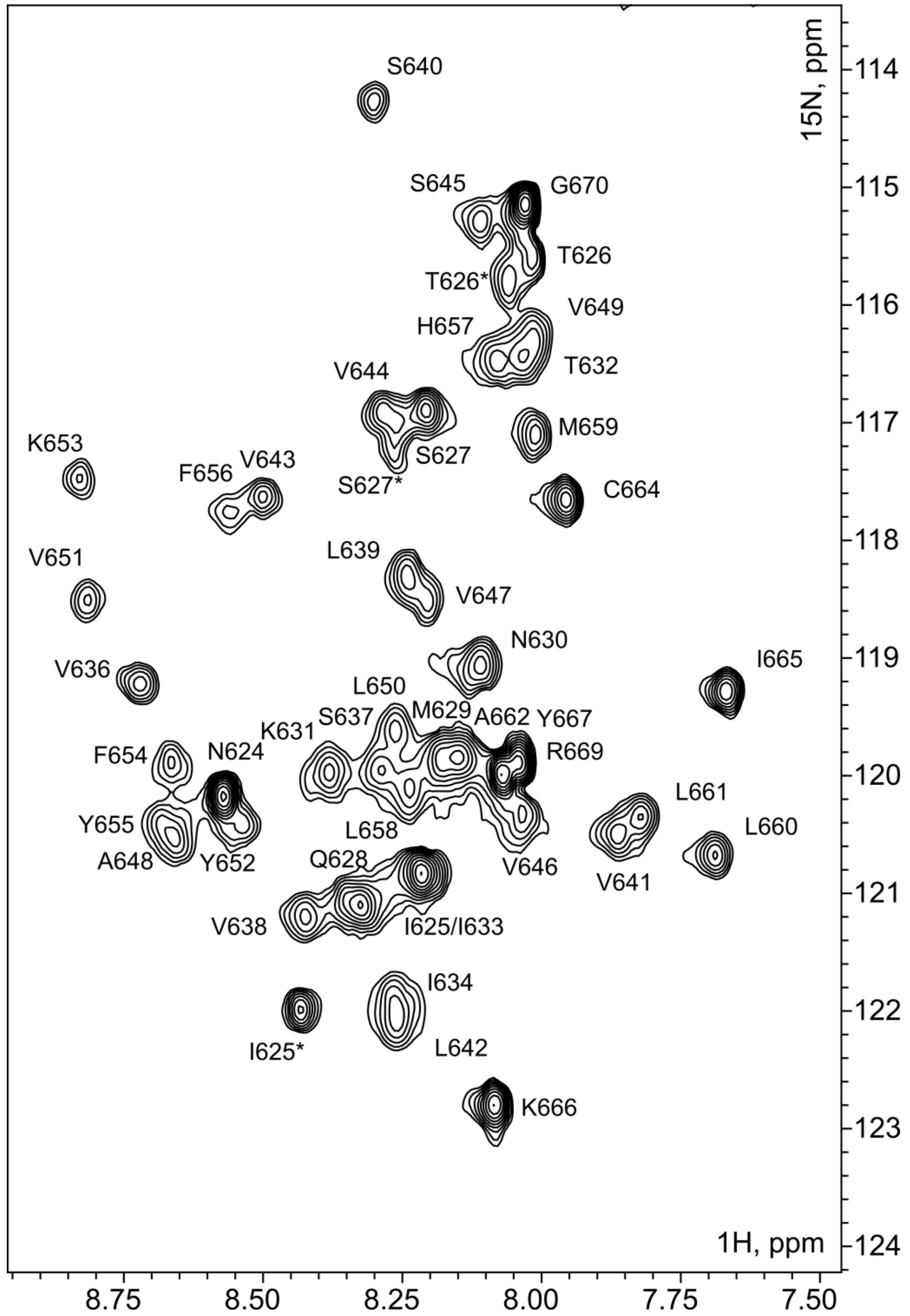

Figure S1B. Refers to Figure 1. Fragment of 1H,15N-BEST-TROSY spectrum of 0.5 mM TLR4-TMICL in DMPC/DHPC q=0.4 bicelles, recorded at pH 6.0, 40 ºC, LPR 200. * denotes the minor state which is present at the N-terminus of the protein due to the partial formylation of the N-terminal residue.

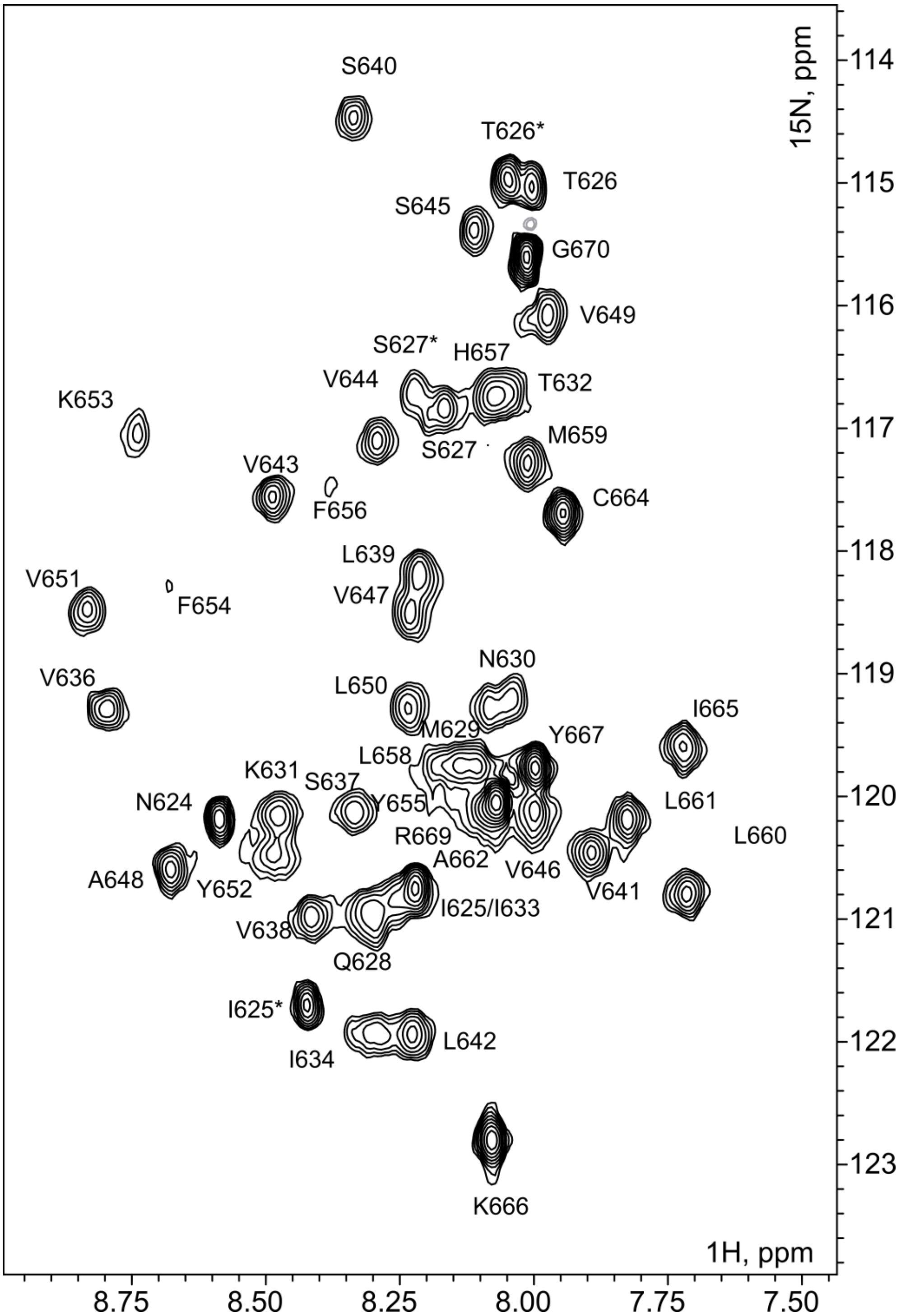

Figure S1C. Refers to Figure 1. Fragment of 1H,15N-BEST-TROSY spectrum of 0.5 mM TLR4-TMICL in DMPG/DHPC q=0.4 bicelles, recorded at pH 6.5, 40 ºC, LPR 200. * denotes the minor state which is present at the N-terminus of the protein due to the partial formylation of the N-terminal residue.

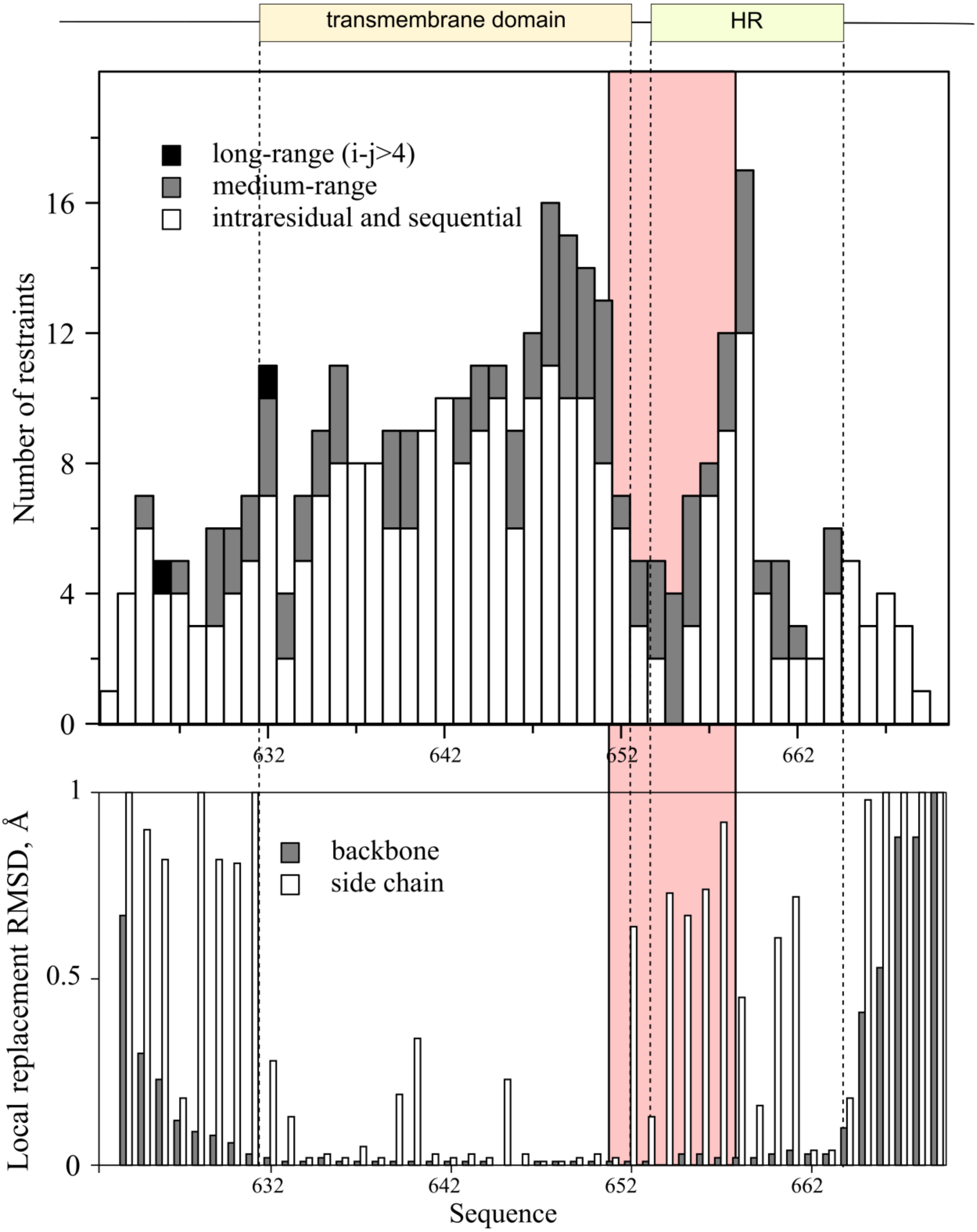

Figure S2. Refers to Figure 2. Statistics for the calculated set of spatial structures of TLR4-TMICL in DMPG/DHPC bicelles. Upper panel shows the number of NOE contacts observed for each residue of TLR4-TMICL. White bars stand for intraresidual and sequential contacts, gray bars - for medium-range contacts and black bars – for long-range contacts. Lower panel shows the local replacement backbone (gray bars) and side-chain (white bars) RMSD relative to the mean structure, as calculated by MOLMOL software in the set of 10 NMR structures of TLR4-TMICL. The discussed in the main text region 652-657 is highlighted by red. RMSD values greater than 1 Å are cut.

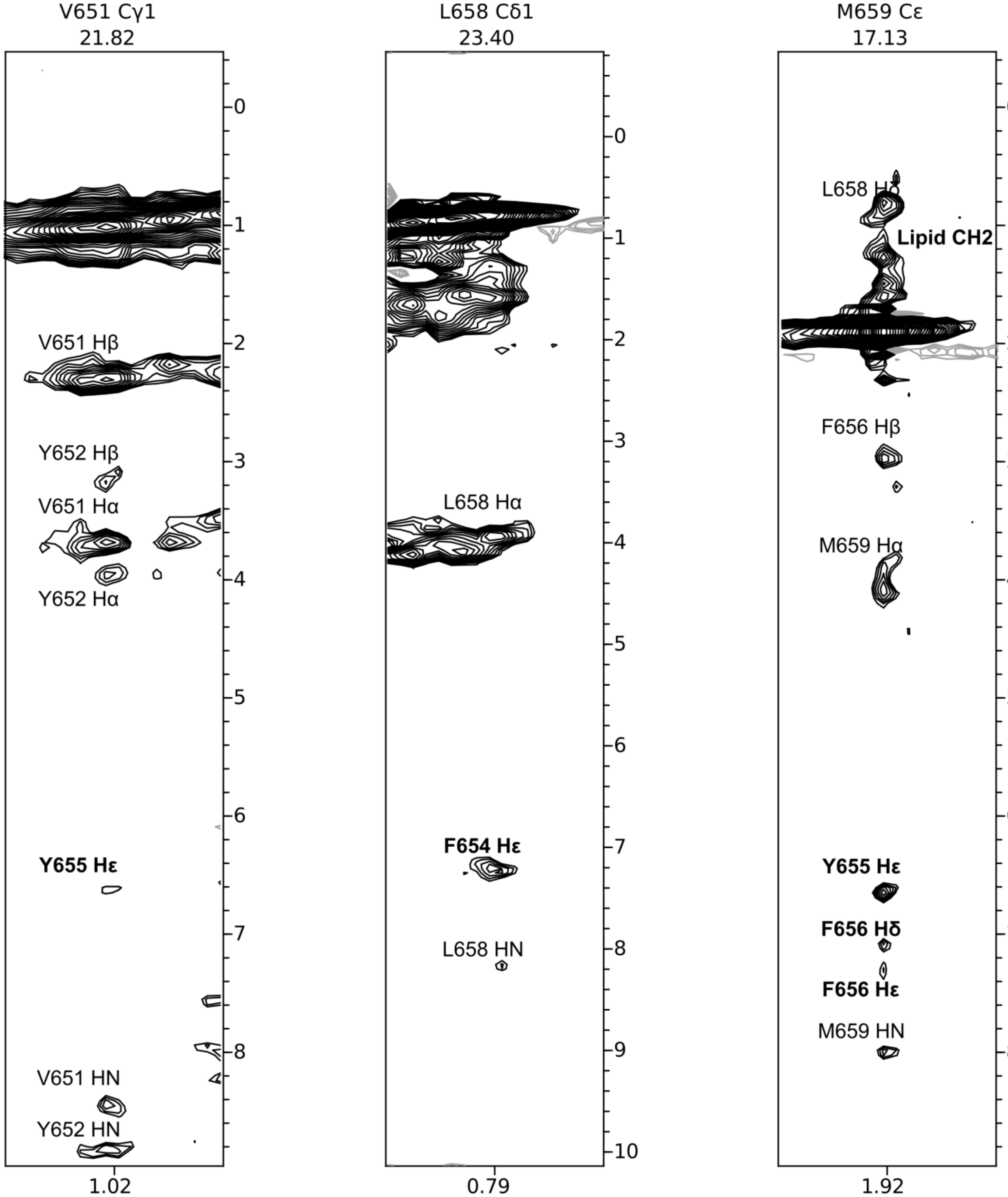

Figure S3. Refers to Figure 2. 2 D **s**trips from the 3D 1H,13C-NOESY-HSQC spectrum of TLR4-TMICL in DMPG/DHPC q=0.4 bicelles, recorded at pH 6.5, 40 ºC, LPR 200. Strips correspond to the signals from the methyl groups of residues V651, L658 and M659. Assignments of NOE contacts are shown.

Table S1. Statistics for the input NMR data and results of spatial structure calculations for TLR4-TM and TLR4-TMICL.

**NMR distance & dihedral restraints**		
TLR4 construct	TMICL	TM
Total unambiguous NOE restraints	206	225
intra-residue	64	95
inter-residue	143	130
sequential (|i-j|=1)	101	53
medium-range (1<|i-j|≤4)	42	77
long-range (|i-j|>4)	0	0
Hydrogen bond restraints (upper/lower)	60/60	63/63
Total torsion angle restraints	85	62
backbone φ	33	25
backbone ψ	33	25
side chain χ^1	19	12
**Structure calculation statistics**		
CYANA target function* (Å^2)	1.05±0.08	0.84±0.15
Restraint violations*		
distance (>0.2 Å)	4*	3
distance (>0.3 Å)	0	0
dihedral (>5°)	0	0
Average pairwise RMSD (Å)		
α-helical region	631-663	631-656
backbone atoms	0.40±0.13	0.34±0.21
all heavy atoms	0.86±0.12	0.95±0.19
Ramachandran analysis**		
% residues in most favored regions	88.4	90.6
% residues in additional allowed regions	11.6	9.4
% residues in generally allowed regions	0	0
% residues in disallowed regions	0	0

* violated restraints refer to the terminal residues of the protein with high intramolecular mobility and appear due to the high dynamic range and a large number of broad residues with weak cross-peak in NOESY that affect the calibration

**Ramachandran analysis and helix-helix contact area calculations were performed with PDBsum (http://www.ebi.ac.uk/pdbsum/)

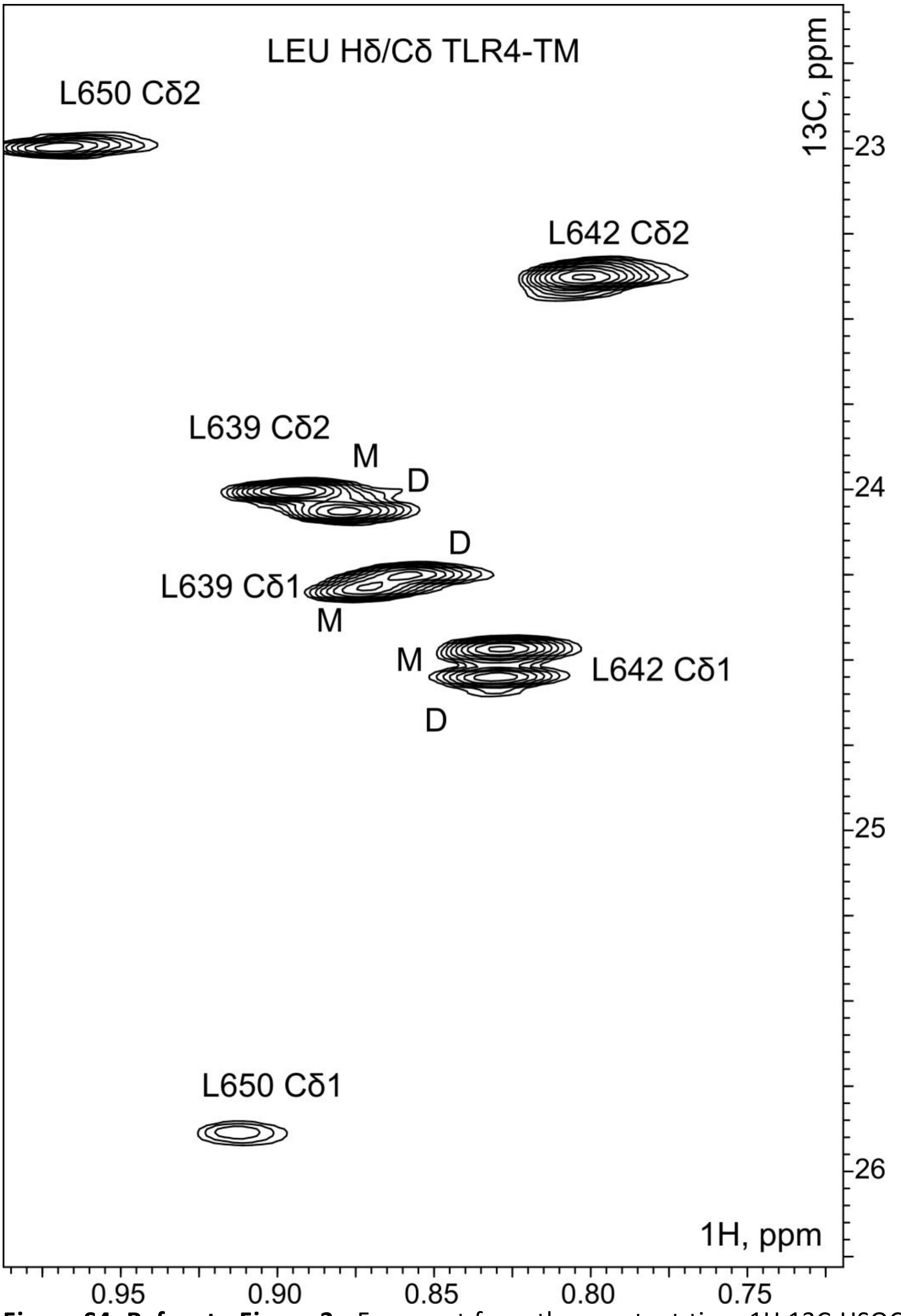

Figure S4. Refers to Figure 3. Fragment from the constant-time 1H,13C-HSQC spectrum of TLR4-TM (pH 6.0, 40 ºC, LPR 60). Fragment contains all signals form the protein methyl groups of Leu sidechains. Assignment of signals to monomeric and dimeric states is indicated by letters M and D, respectively. Constant-time delay was equal to 84.9 ms to obtain the maximal resolution, while retaining the sensitivity.

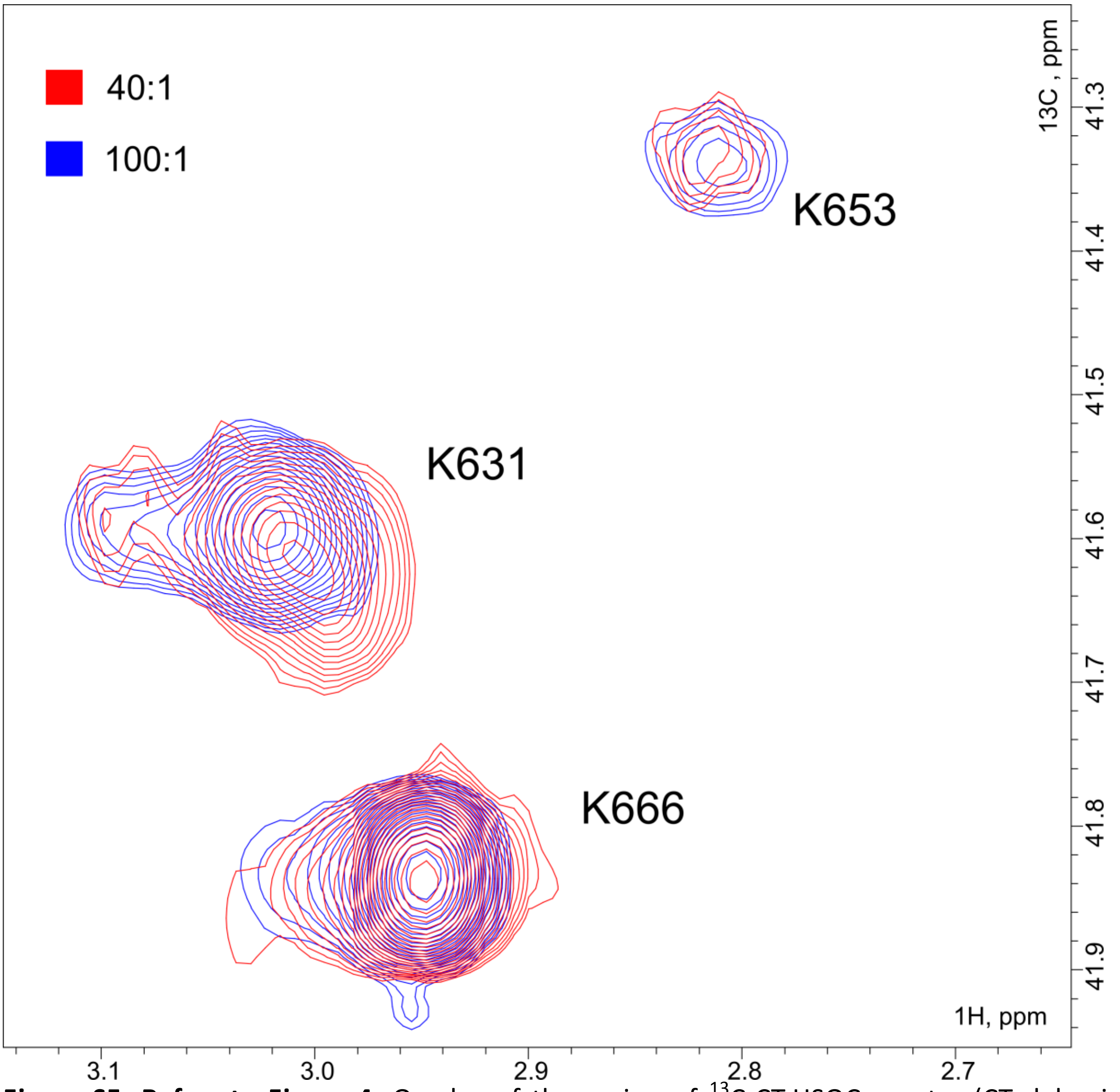

Figure S5. Refers to Figure 4. Overlay of the region of ^{13}C-CT-HSQC spectra (CT delay is equal to 56.6 ms) of TLR4-TMICL in DPC micelles, recorded at LPR 40 (dimer-monomer equilibrium, shown in red) and 100 (pure monomer, shown in blue). Region contains the cross-peaks, corresponding to CεHε groups of lysine residues; assignment of cross-peaks is indicated. No chemical shift changes are observed for the K653 side-chain upon the dimerization, which contradicts the Model 2 (Figure 4B in the main text). At the same time, side-chain of K631, which is at the N-terminus of TM α-helix, reveals the slightly perturbed chemical shifts due to the dimerization of TLR4-TMICL.
